# Alpha-Particle-Induced Complex Chromosome Exchanges Transmitted through Extra-Thymic Lymphopoiesis *In Vitro* Show Evidence of Emerging Genomic Instability

**DOI:** 10.1371/journal.pone.0134046

**Published:** 2015-08-07

**Authors:** Natalia Sumption, Dudley T. Goodhead, Rhona M. Anderson

**Affiliations:** 1 Medical Research Council, Didcot, Oxon, United Kingdom; 2 Division of Biosciences, Department of Life Sciences, College of Health and Life Sciences, Brunel University London, Uxbridge, United Kingdom; University of Oxford, UNITED KINGDOM

## Abstract

Human exposure to high-linear energy transfer α-particles includes environmental (e.g. radon gas and its decay progeny), medical (e.g. radiopharmaceuticals) and occupational (nuclear industry) sources. The associated health risks of α-particle exposure for lung cancer are well documented however the risk estimates for leukaemia remain uncertain. To further our understanding of α-particle effects in target cells for leukaemogenesis and also to seek general markers of individual exposure to α-particles, this study assessed the transmission of chromosomal damage initially-induced in human haemopoietic stem and progenitor cells after exposure to high-LET α-particles. Cells surviving exposure were differentiated into mature T-cells by extra-thymic T-cell differentiation *in vitro*. Multiplex fluorescence *in situ* hybridisation (M-FISH) analysis of naïve T-cell populations showed the occurrence of stable (clonal) complex chromosome aberrations consistent with those that are characteristically induced in spherical cells by the traversal of a single α-particle track. Additionally, complex chromosome exchanges were observed in the progeny of irradiated mature T-cell populations. In addition to this, newly arising *de novo* chromosome aberrations were detected in cells which possessed clonal markers of α-particle exposure and also in cells which did not show any evidence of previous exposure, suggesting ongoing genomic instability in these populations. Our findings support the usefulness and reliability of employing complex chromosome exchanges as indicators of past or ongoing exposure to high-LET radiation and demonstrate the potential applicability to evaluate health risks associated with α-particle exposure.

## Introduction

Ionising radiation deposits energy in the form of tracks of ionisations and excitations that vary in spatial structure depending on the type or quality of radiation [1, 2]. A useful quantity to distinguish these tracks is the linear energy transfer (LET; expressed in units of keV/μm) which specifies the average energy transferred per unit length of the track and which correspondingly differentiates sparsely (e.g. x-rays, γ-rays) from densely (e.g. α-particles, neutrons) ionising radiations as low and high-LET radiation respectively. High-LET α-particles emitted during natural radioactive decay have short ranges (~20–80 μm in body tissue) so are poorly penetrating limiting their relevance for human health risks unless the radioactive material is inhaled, ingested or otherwise internalized within the body. For most radiobiological effects, α-particles are considerably more effective per unit absorbed dose than are low-LET radiations [1, 3] and for radiation protection purposes a radiation weighting factor of 20 is applied [4, 5]. Sources of human exposure to high-LET α-particles include radon gas and its short-lived decay products in the environment, natural α-particle-emitting radionuclides ingested in food, α-particle-emitting radionuclides administered for therapeutic purposes [6, 7] and, occupational contaminants such as plutonium in the nuclear industry [8]. Occupational exposure to radon and its short-lived decay products has been associated with the development of lung cancer in Uranium miners [3, 8–10] while a collaborative analysis of European case-control studies has shown significant association between lung cancer and exposure to radon and its progeny in homes estimating that residential radon is responsible for about 2% of all deaths from cancer in Europe [11, 12]. Exposure to radon is also thought to be relevant in a proportion of environmentally induced leukaemias [3, 13, 14]. Assuming that the relative biological effectiveness of α-particles for leukaemogenesis is 20, in accordance with the radiation weighting factor, it can be estimated that about 7% of leukaemias in young people (to age 25) are attributable to natural high-LET radiation, mostly from ingested and inhaled α-particle emitters [15]. The current risk estimates for leukaemogenesis due to α-emitting radionuclides, including bone seeking radionuclides such as ^223^Ra, however remain uncertain principally due to the non-uniform dose distribution of α-particles *in vivo* and uncertainties in the bone marrow distribution of target cells for leukaemia induction [16].

To further understanding of α-particle effects implicated in leukaemogenesis and also to seek general markers of individual exposure to α-particles, we have been investigating the characteristic damage induced by α-particles using the technique of multiplex *in situ* hybridization (M-FISH), which allows genome-wide resolution of inter-chromosomal damage [17, 18]. Complex chromosome aberrations (rearrangements involving two or more chromosomes with three or more breaks) [19] have been shown to be induced effectively in a range of cell types after exposure to high-LET α-particles both *in vitro* and *in vivo* [20–26] and may be useful as reliable indicators of α-particle exposure since their characteristic complexity can be mechanistically correlated to the interaction between the α-particle track structure and the nuclear organisation of the cell type exposed [1, 27–31]. Additionally, background levels of complex chromosome aberrations in normal populations are extremely low and they are not induced at detectable levels after exposure to low doses of low-LET radiations [20, 21, 32]. Due to their structural complexity, the majority of α-particle-induced complex exchanges induced in peripheral blood lymphocytes (PBL) are non-transmissible through cell division however ~1–2% are capable of long-term persistence [33, 34]. If the same types of transmissible damage are induced also in cells without a finite lifespan, such as the hierarchical stem cells (HSC) and bone marrow (BM) progenitors of lymphocytes, then this could be useful as a lifetime indicator of past and ongoing α-particle exposure. Indeed results from a related study show that complex chromosome aberrations are induced in human CD34^+^ bone marrow progenitor populations consistent with those observed in PBL after exposure to a fluence of ~1 high-LET α-particle/cell [29] and, that a proportion of these aberrations were transmissible over 73 hours *in vitro* culture.

The aim of this study was to assess the transmissibility of complex chromosome exchanges through T-lineage differentiation over several cell generations from BM HSC/progenitors into mature T-lymphocytes *in vitro*. An additional aim was to monitor the cytogenetic changes through cell division to ask questions on the genomic stability of the progeny of irradiated BM cells. For this purpose, a T-cell differentiation culture system, adapted from methodologies described by Ahmad *et al*., 1986, was established [35]. T-cell maturation is based on the reproduction of an extrathymic developmental pathway known to operate *in vivo* in both athymic mice and humans. T-cell (CD3^+^) immunodepleted human bone marrow mononuclear cells (BMNC) (Td) were stimulated for new T-lymphopoiesis (CD3^+^/CD4^+^, CD3^+^/CD8^+^) in this culture system following irradiation with a mean of 1 α-particle traversal/cell (0.5 Gy; incident LET 121 keV/μm, assumed cell diameter 7 μm). Subsequent transfer of naïve T-cell populations into bulk culture for expansion of new T-cell numbers enabled chromosomal damage, including clonal complex aberrations characteristic of high-LET radiation exposure, to be assessed by M-FISH. We found stable complex chromosome aberrations consistent with those characteristically induced by the traversal of a single α-particle track in naïve T-cell populations, supporting the usefulness of employing complex chromosome exchanges as indicators of past or ongoing exposure to high-LET radiation. We also observed evidence of genomic instability in cells carrying such clonal markers of α-particle exposure and also in cells that did not which may provide insight into possible mechanisms for radiation-induced genomic instability.

## Materials and Methods

### Immunodepletion of T-lymphocytes

Fresh human bone marrow mononuclear cells (BMNC) (Cambrex, US; Food and Drug Administration (FDA) approved tissue bank) from two healthy adult donors (never smokers) were obtained commercially with full donor consent and shipped for receipt within 24 hours of collection. The research was approved by the MRC Harwell Research Ethics Committee and samples were handled in accordance to the guidelines issued by the Medical Research Council in *Responsibility in Investigations on Human Participants and Material and on Personal Information* (MRC Ethics series, November 1992). Following overnight incubation at 37°C, 5% CO_2_ in T75 vented flasks (Greiner, UK), BMNC were immunodepleted of intermediate and mature T-lymphocytes using Easysep^TM^ technology (Stemcell Technologies, UK) as follows. After centrifugation at 200g for 10 minutes, BMNC were resuspended in Easysep^TM^ buffer at a density of 1x10^8^ total cells/ml (maximum volume of 2.5 mls) in round-bottomed 5 ml polystyrene tubes (BD falcon, UK). Easysep^TM^ cell separation cocktails containing monoclonal antibodies directed towards T-cell surface markers CD3 and CD8 were added to buffered cell suspensions (200 μl/ml for each antibody). Simultaneous addition of 0.4 μg/ml of FITC-conjugated monoclonal CD3 antibody (Serotec, UK) allowed the mature T-cell population to be labelled for subsequent analysis by fluorescence activated cell sorting (FACS). After 15 minutes incubation in the dark and at room temperature, cell suspensions were cross-linked to 100 μl/ml Easysep^TM^ magnetic colloid nanoparticles and incubated for a further 10 minutes. For immunodepletion, cell suspensions were placed within a magnetic block for 10 minutes. T-cells bound to the antibody:colloid complex were held by the magnetic field, whilst unattached cell fractions were poured off in supernatant allowing T-positive (Tp) and T-depleted (Td) samples to be independently collected for culture. The depletion process was repeated on separated Td populations to ensure a near maximal 3–4 log T-cell reduction. A sample of undepleted whole bone marrow mononuclear cells (Tw) was retained for use as a positive culture control. Immediately after depletion, Td, Tw and Tp samples were irradiated.

### Irradiation and cell culture

Separate Td, Tp and Tw samples were plated into Hostaphan dishes (2x10^6^ cells/dish) for sham or α-particle irradiation; 8 μls of monolayer cell suspension was overlaid with a CR39 plastic disc and exposed to α-particles using a ^238^Pu irradiation source at room temperature [21, 36]. Incident energy of the α-particles entering the cells at the Hostaphan base of the dish was 3.26 MeV (LET of 121.4 keV/μm) and the dose delivered was 0.5 Gy (dose rate 1.5 Gy/min) (corresponding to a mean of 1 α-particle traversal/cell (0.67 tracks/nucleus) for cells of assumed average diameter 7 μm or projected area 40 μm^2^) [21, 29].

Directly following irradiation, sham and α-particle exposed (Td, Tp and Tw) populations were washed from the Hostaphan dish (recovery ~90%) and seeded in either the presence or absence of T-cell stimulatory factors. Specifically, samples were seeded at 2x10^5^ cells/ml in 200 μl volumes of basic media (RPMI 1640 Dutch modification (Invitrogen, UK), containing 20% human pooled AB serum (Mast, UK), 100 IU/ml penicillin, 100 μg/ml streptomycin, 2mM L-glutamine, and 2mM sodium pyruvate (Sigma-Aldrich, UK)). Td fractions, and Tp and Tw positive controls were stimulated for T-lymphocyte differentiation and growth by addition of 0.5 μg/ml purified phytohaemagglutinin (PHA: HA16, Bio-Stat Ltd, UK) and 100 IU/ml recombinant IL-2 (R&D systems, UK) every four days throughout the culture period. Stimulated samples were supplemented with 25% conditioned media (described below) on day 0. Negative Td, Tp and Tw control populations received no T-cell stimulation. All samples were cultured for 15 days in 96 well microtitre plates (Greiner, UK), incubated at 37°C, 5% CO_2_.

For T-cell expansion, samples were transferred to a long-term lymphocyte culture system [33]. Td, Tp and Tw populations (excluding negative controls) were grown in bulk at 1x10^5^ cells/ml in 4 ml volumes of fresh culture media (basic media plus T-cell stimulatory factors 0.5 μg/ml purified phytohaemagglutinin (PHA: HA16, Bio-Stat Ltd, UK) and 100 IU/ml recombinant IL-2 (R&D systems, UK)). Lethally irradiated feeders (human lymphoblastoid cells exposed to 40 Gy of 250 kV X-rays) were added at a concentration of 5x10^4^ cells/ml. Culture media and feeders were refreshed every four days to maintain continuous T-cell proliferation over two to three weeks. To enable collection of metaphase cells for M-FISH analysis, 5 μl/ml colcemid (Sigma-Aldrich, UK) was added to growing cell fractions for the final 1.5 hours of culture. Cells were then treated with hypotonic buffer (1:1 0.075M KCl:0.8% NaCit in dH_2_0) at 37°C for 9 minutes prior to fixation in 3:1 methanol:acetic acid and storage in suspension at –20°C.

### Conditioned media

Conditioned media was prepared according to the method described by Ahmad *et al*. [35]. The research was approved by the MRC Harwell Research Ethics Committee and whole blood was collected according to the guidelines issued by the Medical Research Council in *Responsibility in Investigations on Human Participants and Material and on Personal Information* (MRC Ethics series, November 1992). Blood samples were immunodepleted of T-cells using Rosettesep technology (Stemcell Technologies,UK). Rosettesep T-cell depletion cocktails containing monoclonal antibodies CD3 and CD8 were mixed (50 μl/ml of each) with whole blood prior to peripheral blood mononuclear cell (PBMNC) isolation based on Ficoll density gradient centrifugation using sodium heparin Vacutainer tubes (Becton Dickinson, UK). Resulting non-T PBMNC fractions were washed in CaCl_2_/MgCl_2_ free phosphate buffered saline (PBS) (Sigma-Aldrich, UK). Pooled cells were seeded at 1x10^6^/ml in 4 ml volumes of RPMI 1640 media (Dutch modification (Invitrogen, UK)) containing 2% heat-inactivated pooled human AB serum (Mast, UK), 0.5 μg/ml purified phytohaemagglutinin (PHA: HA16, Bio-Stat Ltd, UK), 100 IU/ml penicillin, 100 μg/ml streptomycin, 2mM L-glutamine, and 2mM sodium pyruvate (Sigma-Aldrich, UK). T-depleted PBMNC populations were incubated at 37°C, 5% CO_2_ for 3 days before collection and –20°C storage of filtered cell supernatants (Minisart 0.2 μM filters, Sartorius Ltd, UK) for use as conditioned media.

### Immunofluorescence

Heterogeneous Td, Tw and Tp population profiles were characterised throughout the culture period using a panel of anti-human monoclonal fluorescence conjugated antibodies (Serotec, UK). Specifically, T-cell depletion, differentiation and repletion were assessed every three days with fluorescence activated cell sorting (FACS) measurement of CD3, CD4 and CD8 T-cell surface marker expression levels. The presence of non-T cell types was monitored to day 9 using CD14, CD33, CD66b, CD42b, CD235a, CD19 and CD56 antibodies to detect the presence of intermediate/mature monocytes and macrophages, intermediate myeloid cells and mature monocytes, mature granulocytes, platelets and megakaryocytes, red cells, B cells, and natural killer cells respectively. Isotypes were IgG1, IgG2b mouse and IgG2b rat negative controls.

For each timepoint investigated, a minimum of 2x10^4^ viable cells/antibody were aliquoted for labelling into 100 μl cold CaCl_2_/MgCl_2_ free phosphate buffered saline (PBS) (Sigma-Aldrich, UK) containing 1% bovine serum albumin fraction V (7.5% BSA) (Sigma-Aldrich, UK) and 20 mM glucose (Becton Dickinson, UK). Cell samples were incubated in the dark for 30 minutes at 4°C with 10 μl of neat antibody (20 μl for CD66b). Cells were then washed with 1 ml of fresh cold PBS/BSA/glucose solution to remove excess antibody and were diluted into 500 μl PBS for immediate analysis on an argon laser Becton Dickinson FACSort. Antibody-labelled samples were kept in the dark on ice until FACS analysis was completed. When immediate analysis was impractical, samples were fixed in 500 μl 1:10 cellfix:PBS (Becton Dickinson, UK; Sigma-Aldrich, UK) and stored at 4°C for no more than three days before FACS measurements were recorded. Results were produced in histogram and/or dotplot format based on calculation of percentage shifts in single or dual fluorescence (FITC/PE) relative to isotype controls. Where distinct subpopulations were observed, specific gating allowed a more accurate assessment of the size of T-cell fractions within Td, Tw and Tp populations.

Direct slide immunofluorescence was also carried out. Antibody-labelled cells were pelleted and resuspended in 10 μl PBS/BSA/glucose solution (as previously described), then mixed with 10 μl vectashield containing DAPI counterstain (Vector Laboratories, UK) and 0.1 μl of 2mg/ml propidium iodide (Molecular Probes, UK) for exclusion of non-viable cells (PI^+^) from the analysis. Cells were dropped onto slides, overlayed with a coverslip and scored for FITC and/or Cy-5 fluorescence.

### Detection of T-cell receptor excision circles (TRECs)

Naïve T-cell production in Td populations was assessed throughout differentiation culture phase, from initial T-cell depletion to emergence of new CD3^+^ expressing cells (days 0–12). During this period, a minimum of 1x10^4^ cells (but where possible 1x10^6^ cells), were collected for real-time PCR analysis of TREC levels at 2/3 day intervals. At each time-point, Td samples were washed in CaCl_2_/MgCl_2_ free phosphate buffered saline (PBS) (Sigma-Aldrich, UK), and cell pellets incubated at 55°C in 25 μl of lysis buffer (1 M Tris, 0.5 M EDTA, 10% Tween containing 5% 10 mg/ml proteinase K) (Sigma-Aldrich, UK). The lysis reaction was inactivated after two hours with a 5–10 minute incubation at 95–100°C. All undigested cellular debris was removed from resulting DNA lysates by centrifugation so that only lysate supernatants were used in PCR. Real-time PCR for generation of TREC measurements was kindly performed by Lena Al-Harthi and John P. Voris at the Rush University Medical Center in Chicago, US [37]. The following calculation was used to express assay data in terms of TRECs/10^5^ cells:

$$\frac{Total\;cells\;in\;lysate}{Total\;lysate\;volume\;(25\;ul)\times lysate\;volume\;run\;in\;PCR\;(2\;ul)\;}\;=\;Total\;cells\;assayed$$

$$\frac{Number\;of\;TRECs\;detected\;\times 1\times 10^{5}}{Number\;of\;cells\;assayed\;}\;=\;Number\;of\;TRECs/10^{5}cells$$

### Multiplex fluorescence *in situ* hybridization (M-FISH)

For M-FISH, slides were left at room temperature for 24 hours to harden, before aging in fixative for 1 hour (3:1 methanol:acetic acid), incubation at 67°C for 20 minutes, and immersion in acetone for 10 minutes. Hybridisation pretreatments were carried out at 37°C, consisting of 1 hour incubation in 100 mg/μl DNAse free RNAse (Sigma, UK) and 5–10 minutes digestion in pepsin (Sigma, UK) (1:20x10^3^ in 10 mM HCL). Slides were rinsed twice in 2xSSC and PBS between pretreatments, and twice in PBS following pretreatments (for 5 minutes each). Additional washes in 50 mM MgCl in PBS, and 50 mM MgCl in PBS with 1% formaldehyde (5 minutes each) preceded slide dehydration through an ethanol series (70, 70, 90, 90, 100% ethanol for 2 minutes each).

For hybridisation, probe cocktail (24-colour paint Spectravision assay, Vysis Ltd UK) was denatured at 72°C for 6 minutes. In parallel, metaphase chromosomes, immersed in 70% formaldehyde/2xSSC, were denatured at 72°C for 3 minutes before being dehydrated through an ethanol series (70, 90, 100% for 1 minute each) and air-dried. Probe was then hybridised to cells during a 48–72 hour incubation period at 37°C. For post-hybridisation washes, slides were rinsed in 0.4xSSC/0.3% Igepal (Sigma, UK) for 1.5 minutes at 71°C and 2xSSC/0.1% Igepal for 10 seconds at room temperature to remove unhybridised probe. A DAPI III counterstain (Vysis Ltd, UK) was applied to air-dried slides, coverslips sealed and cells stored in the dark at -20°C.

Metaphase chromosomes were visualized using a six-position Olympus BX51 fluorescence microscope containing individual filter sets for each component fluor of the Spectravision (Vysis Ltd, UK) probe cocktail plus DAPI (Spectrum Gold, Spectrum Far-red, Spectrum Aqua, Spectrum Red and Spectrum Green). Digital images were captured for M-FISH using a charge-coupled device (CCD) camera (Photometrics Sensys CCD) coupled to and driven by Genus (Applied Imaging, UK). In the first instance, cells were karyotyped and analysed by enhanced DAPI banding. Detailed paint analysis was then performed by assessing paint coverage for each individual fluor down the length of each individual chromosome, using both the raw and processed images for each fluor channel. A cell was classified as being apparently normal if all 46 chromosomes were observed by this process and subsequently confirmed by the Genus M-FISH assignment, to have their appropriate combinatorial paint composition down their entire length.

### Analysis of chromosome and chromatid-type aberrations

Metaphase cells were harvested from Td, Tw and Tp populations during expansion culture phase (time-points between days 15 and 35) and analysed by M-FISH using coded slides. All observed chromosomal damage was categorized for aberration type as follows: exchange aberrations involving 3 or more breaks in 2 or more chromosomes were classed as complex; rearrangements involving a maximum of 2 breaks in 2 chromosomes were classed as simple; un-restituted free ends not apparently involved in an exchange of material between chromosomes were classed as single chromosome breaks. Chromatid breaks were also recorded, as well as aneuploid changes (full and partial chromosome gains/losses).

Complex and simple exchanges were designated as transmissible or non-transmissible based on the absence/presence of dicentrics/acentric fragments within each rearrangement event (for detail see [33]).

Chromosome aberrations were established as clonal where two or more metaphases with the same structural anomaly or extra chromosome were observed. To avoid overestimation of induced damage, groups of cells displaying an identical clonal aberration, assumed to have derived from one ancestral cell, were scored as single damage events. Within each clone, all newly arising aberrations, whether of chromosome or chromatid type, were scored to assess ongoing karyotypic evolution.

## Results

### Differentiation and maturation of lymphoid cells via an extrathymic pathway

A model for *in vitro* lymphopoietic culture of irradiated human bone marrow mononuclear cells (BMNC) was optimised. Each element of the methodology was developed over a number of different experiments using several different donors. Two independent experiments were subsequently carried out to differentiate sham and α-particle irradiated haemopoietic stem and progenitor cells into T-lymphocytes for cytogenetic sampling.

### Evidence for immunodepletion of intermediate/mature T-lymphocytes

**Fig 1:**
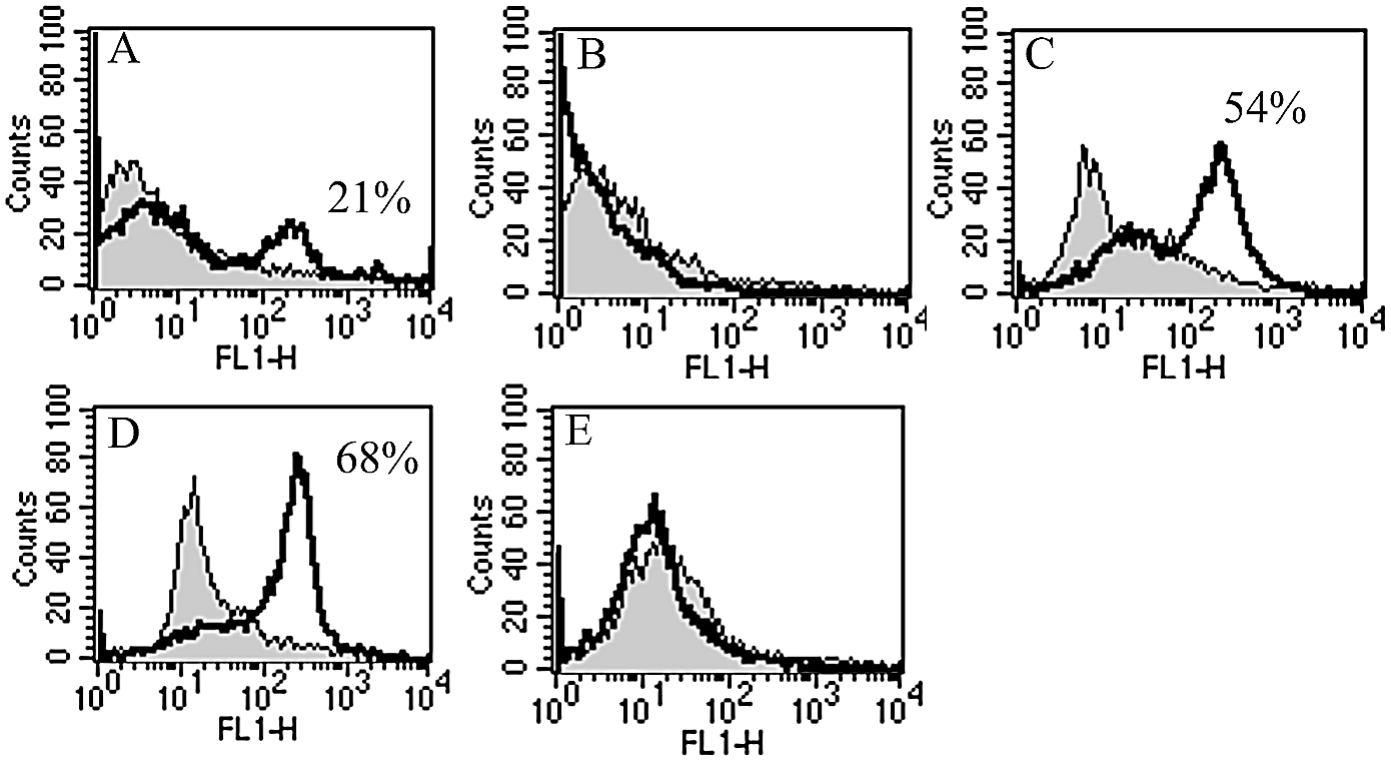
Representative histogram plots show CD3 expression levels measured by FACS. The percentage shift in CD3-FITC fluorescence (overlaid as a black line) was calculated relative to isotype controls (coloured grey). The histogram peaks are typically broad due to the population being of mixed cell type. CD3 expression levels before T-cell depletion of BMNC (Tw) show a mature T-lymphocyte population of 21% (A). Complete depletion of CD3^+^ cells was observed in T-cell depleted (Td) fractions on day 0 (B). A new T-cell subpopulation, accounting for 54% of Td sample, was produced by day 12 (C). Td T-cell proportion was increased over 10 days of long-term culture to reach 68% on day 25 (D). No CD3^+^ cells were observed in Td, Tp or Tw populations cultured in the absence of stimulatory factors PHA and IL-2. Td data only shown in (E).

To determine the proportion of CD3^+^ mature T-cells in Tw and Td populations, FACS analysis of fresh human BMNC was carried out before and after immunodepletion of CD3^+^ and CD8^+^ cells. 21% of Tw populations were CD3^+^ (representing 0.3x10^5^ total cells (Fig 1A)). This decreased to undetectable levels i.e. <1% CD3^+^ cells, in Td samples after immunodepletion (Fig 1B).

TREC levels were also assayed immediately pre- (Tw) and post- (Td) immunodepletion. Tw populations contained 317.3 copies of TREC/10^5^ cells. Following the removal of mature T-cell fractions (Tp), TREC copy number in Td populations was reduced to 2.1/10^5^ cells (representing <1% of original TREC levels). In contrast, separated Tp samples contained enriched TREC levels (2499.7 copies of TREC/10^5^ cells).

### Evidence for repletion of mature T-lymphocytes

A number of different endpoints (cell counts, FACS detection of T and non-T cell surface markers, TREC levels, and T-cell mitogen response) were assessed to investigate whether T-lymphocytes accounted for the repletion in growing Td populations. Initially, total viable cell number in PHA-stimulated Td samples decreased 9–17 fold between days 0 and 12 of culture (a decrease of 0.5–2.5x10^5^ total cells by day 12). In addition, Td FACS data of non-T-lineage cells from day 0 to 9 suggest loss of mononuclear cell populations (intermediate and mature monocytes, granulocytes, platelets, megakaryocytes and erythrocytes) independent of T-cell stimulatory factors. Since lack of both mitogen response and CD3^+^ expression is consistent with an absence of T-cells after immunodepletion (Fig 1B), the overall reduction in Td cell numbers is likely to be a consequence of myeloid lineage type decline. However by day 12, Td cell proliferation was observed. A simultaneous appearance of TRECs (from 0 copies at day 9 to 381.5 copies/10^5^ cells by day 12) and CD3^+^ marker expression (ranging between 30–54%, and representing 0.2-1x10^5^ cells) (Fig 1C) suggests the presence of mature T-cells at this time-point. Subsequent proliferation during bulk expansion culture produced a 5–9 fold increase in total cell number from day 15 onwards (reaching 3–4.6x10^6^ total cells between days 25–36). An associated rise in CD3^+^ expression levels was observed, accounting for between 50–70% of Td populations (representing 1.8–2.1x10^6^ T-cells) (Fig 1D).

Total viable cell number in PHA-stimulated Tw and Tp samples increased 1–2 fold and ~2 fold respectively by day 6 (versus a 0.6 fold decrease in Td samples), reflecting the presence of mature CD3 T-cell populations. On transfer into long-term culture conditions on day 15 for bulk growth, there was a 6–11 fold increase in total cell numbers by day 25 (representing 4.6–9.1x10^6^ cells), and an associated rise in CD3^+^ expression levels to account for 70–80% of total Tw and Tp populations (representing 3.2–7.3x10^6^ T-cells). No T-cell differentiation or proliferation was observed in Td (Fig 1E), Tw or Tp populations without PHA stimulation.

**Fig 2:**
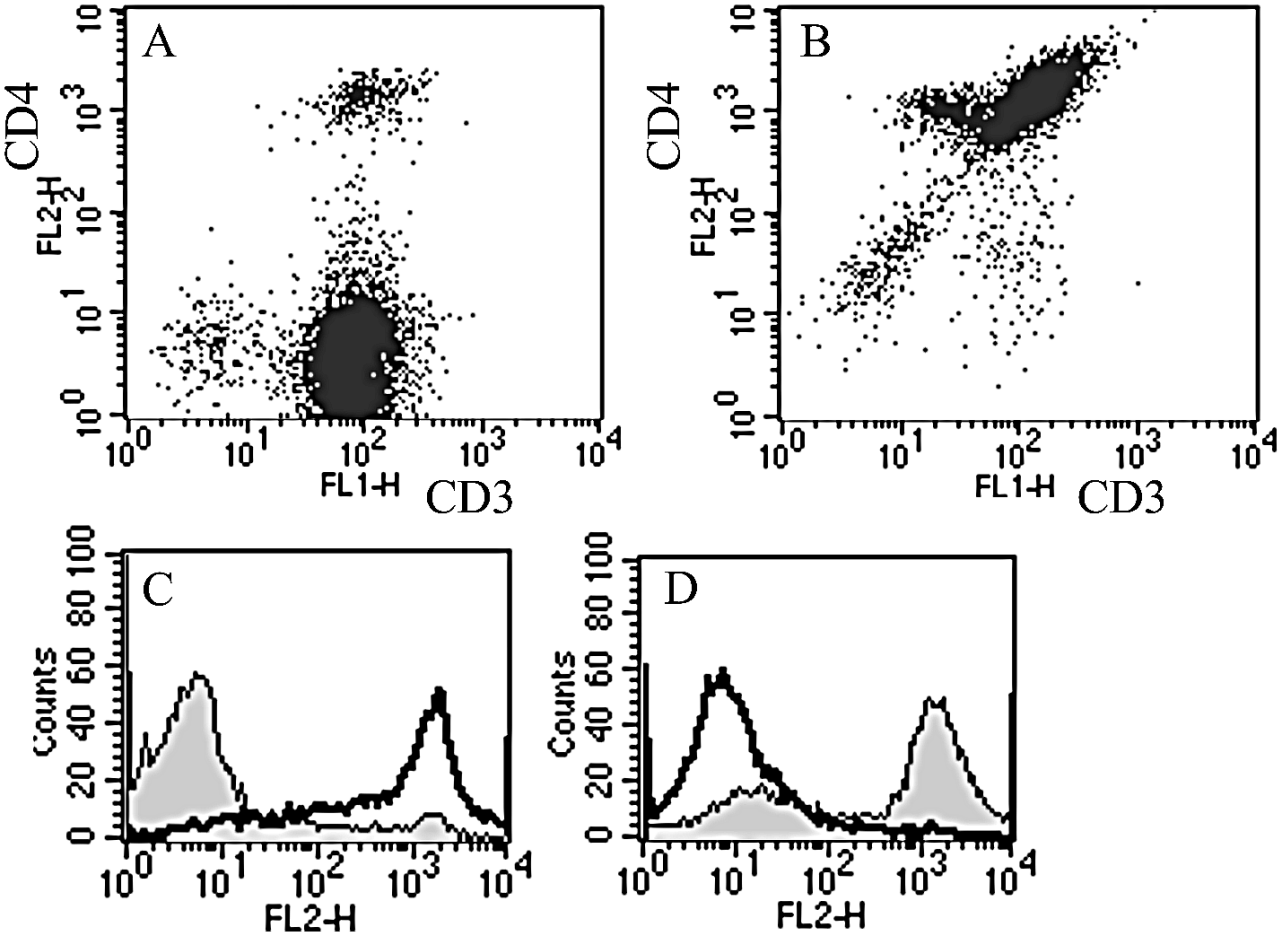
Representative dot-plots showing co-expression measured by FACS. Mononuclear cell sub-populations were gated and representative dot-plots produced showing levels of CD3 and CD4 co-expression measured by FACS. Representative histogram data for the same time-points show CD4 and CD8 expression levels in un-gated populations. The percentage shift in CD4-PE (coloured grey) or CD8-PE fluorescence (overlaid as a black line) was calculated relative to isotype controls (not shown). On day 12, 4% of mature CD3^+^ T-lymphocytes in Tw populations co-express the CD4 cell surface marker (A) compared with 91% in Td populations (B). CD8^+^ cells predominate in Tw samples (C). CD4^+^ cells predominate in Td samples (D).

Levels of CD4 and CD8 T-cell surface markers were assessed alongside CD3 expression to determine the type of mature T-lymphocytes present in each of the three different cultured cell samples (Td, Tw and Tp). Day 12 comparisons of CD3 co-expression with CD4 or CD8 show that Tw and Tp samples are predominantly CD8^+^ (containing 70–88% CD8^+^ versus 14–18% CD4^+^ cells with 2–4% of CD3^+^ cells co-expressing CD4) (Fig 2A and 2B), and Td samples, CD4^+^ (containing 4% CD8^+^ versus 12% CD4^+^ cells with 91% of CD3^+^ cells co-expressing CD4) (Fig 2C and 2D). There was a transient increasing trend in both CD4 and CD8 expression, intermediate T-cell surface markers, prior to CD3 appearance in Td culture (from 0–0.3x10^5^ total cells on day 0 to 0.4–2.2x10^5^ between days 3 and 6, and back to 0–0.2x10^5^ on day 9).

Thus, the coincident appearance of TRECs, cell proliferation, CD4/8 differences and CD3^+^ expression ~ day 12 collectively suggest development of *de novo* T-lymphocytes in immunodepleted BM (Td) when cultured in the presence of PHA and IL-2.

### Application of the extrathymic culture system for cytogenetic assessment of α-particle irradiated T-cell populations

The transmissibility of chromosome aberrations induced in haemopoietic stem and progenitor cells after exposure to high-LET α-particles was examined in new T-lymphocyte populations. Metaphase cells were harvested from Td, Tp and Tw samples for analysis by M-FISH at time-points ranging between 15 and 35 days following irradiation. The data from two independent experiments is presented.

### Chromosome aberrations in *de novo* T-lymphocytes (Td)

Table 1 shows the chromosome aberrations observed in Td samples at varying times following α-particle exposure and lymphopoietic culture. In sham-irradiated populations, no complex exchange aberrations were observed. Similarly, no simple exchanges were detected except for one low level clonal aberration in donor 1 whose presence in both sham and α-particle irradiated fractions (comprising 0.075 and 0.030 of total cells analysed on day 27 respectively) (Table 1 footnote a and b, Table 2) suggests it is unlikely to have been experimentally induced. The aberration was not found at any of the other time-points investigated possibly due to its low incidence and/or dilution with proliferation over time. Similarly for donor 2, a deletion (terminal or interstitial) of 1q formed a clone that was present in both sham (4 cells representing 0.078 of total cells analysed on day 15) and α-particle irradiated fractions (2 cells representing 0.020 of total cells analysed on day 20) (Table 2 footnote a, Table 3 footnote a), again implying this aberration was present prior to set up of these experiments. There was no evidence for any increase in chromosome or chromatid break frequency in sham-irradiated cells with time in culture for either donor (Table 3).

**Table 1:** Chromosome exchange aberrations observed by M-FISH in T-cell depleted bone marrow mononuclear cells exposed to α-particle irradiation.

Exposure	Sample (days)	Total cells	% cells damaged	*f* Complex (no. of cells)		*f* Simple (no. of cells)	
				non-clonal	clonal	non-clonal	clonal
**Donor 1**							
**Sham**	d15	39	8	0	0	0	0
	d27	67	4^a^	0	0	0	0.015^b^ (5)
	d35	48	4	0	0	0	0
**0.5 Gy α-particles**							
	d27	100	5^a^	0	0	0	0.010^b^ (3)
	d35	51	20	0	0	0	0
**Donor 2**							
**Sham**	d15	51	8^a^	0	0	0	0
	d20	27	0	0	0	0	0
	d27	51	14	0	0	0	0
**0.5 Gy α-particles**							
	d15	219	6^a^	0.005	0.005 (58)	0.009^c^ (2)	0
	d20	99	16^a^	0	0.010 (4)	0	0
	d27	90	11	0	0	0	0

^a^ multiple cells (n) with clonal aberration/s scored as 1 cell to ascertain frequency

^b^ clonal damage present in both sham and α-particle irradiated samples

^c^ non-clonal aberration/s within cells containing a clonal aberration

**Table 2:** Evolution of sham and α-particle induced clonal aberrations with time in T-cell depleted bone marrow mononuclear cells.

Exposure	Sample (days)	Total cells (clone)	Clonal karyotype [no. of cells with karyotype]	Evolved karyotypes [no. of cells with karyotype]
**Donor 1**				
**Sham**	d15	39 (0)		
	d27	67 (5^a^)	46,XX,t(8;21)(q1;q1) [5]	
	d35	48 (0)		
**0.5 Gy α-particles**				
	d27	100 (3^a^)	46,XX,t(8;21)(q1;q1) [3]	
	d35	51 (0)		
**Donor 2**				
**Sham**	d15	51 (4^a^)	46,XY,del(1)(q2-qter) [4]	
	d20	27 (0)		
	d27	51 (0)		
**0.5 Gy α-particles**				
	d15	219 (58)	46,XY,t(2;4;17)(p2;q2;q2) [49]	46,idem,t(6;18)(p2;q1) [1]
				46,idem,t(9;14)(q1;q3),+9q [1]
				46,idem,del(2)(q1-qter) [1]
				47,idem,csb(6)(q1:q1) [1]
				46,idem,ctd(3)(q2) [1]
				46,idem,ctd(9)(q1) [1]
				46,idem,ctd(10)(q1) [1]
				47,idem,ctd(12)(q2),+20 [1]
				47,idem,+14 [1]
	d20	99 (4)	46,XY,t(2;4;17)(p2;q2;q2) [3]	47,idem,csb(13)(q2:q2) [1]
		(2^a^)	46,XY,del(1)(q2-qter) [1]	46,idem,del(X)(q2-qter) [1]
		(2^a^)	46,XY,+(8)(pter-q2) [1]	47,idem,+8 [1]
	d27	90 (0)		

^a^ clonal damage present in both sham and α-particle samples / idem; denotes clonal aberration

**Table 3:** Occurrence of non-clonal break aberrations in T-cell depleted bone marrow mononuclear cells exposed to α-particle radiation.

Exposure	Sample (days)	Total cells	Number of breaks (*f*)	
			chromosome	chromatid
**Donor 1**				
**Sham**	d15	39	1 (0.026)	2 (0.051)
	d27	67	3 (0.045)	0
	d35	48	0	2 (0.042)
**0.5 Gy α-particles**				
	d27	100	4 (0.040)	1 (0.010)
	d35	51	2 (0.039)	10 (0.196)
**Donor 2**				
**Sham**	d15	51	3^a^ (0.059)	0
	d20	27	0	0
	d27	51	3 (0.059)	4 (0.078)
**0.5 Gy α-particles**				
	d15	219	6 (0.027)	13 (0.059)
	d20	99	9^a^ (0.091)	4 (0.040)
	d27	90	1 (0.011)	11 (0.122)

^a^ includes clonal aberration scored as 1 cell to ascertain frequency

**Fig 3:**
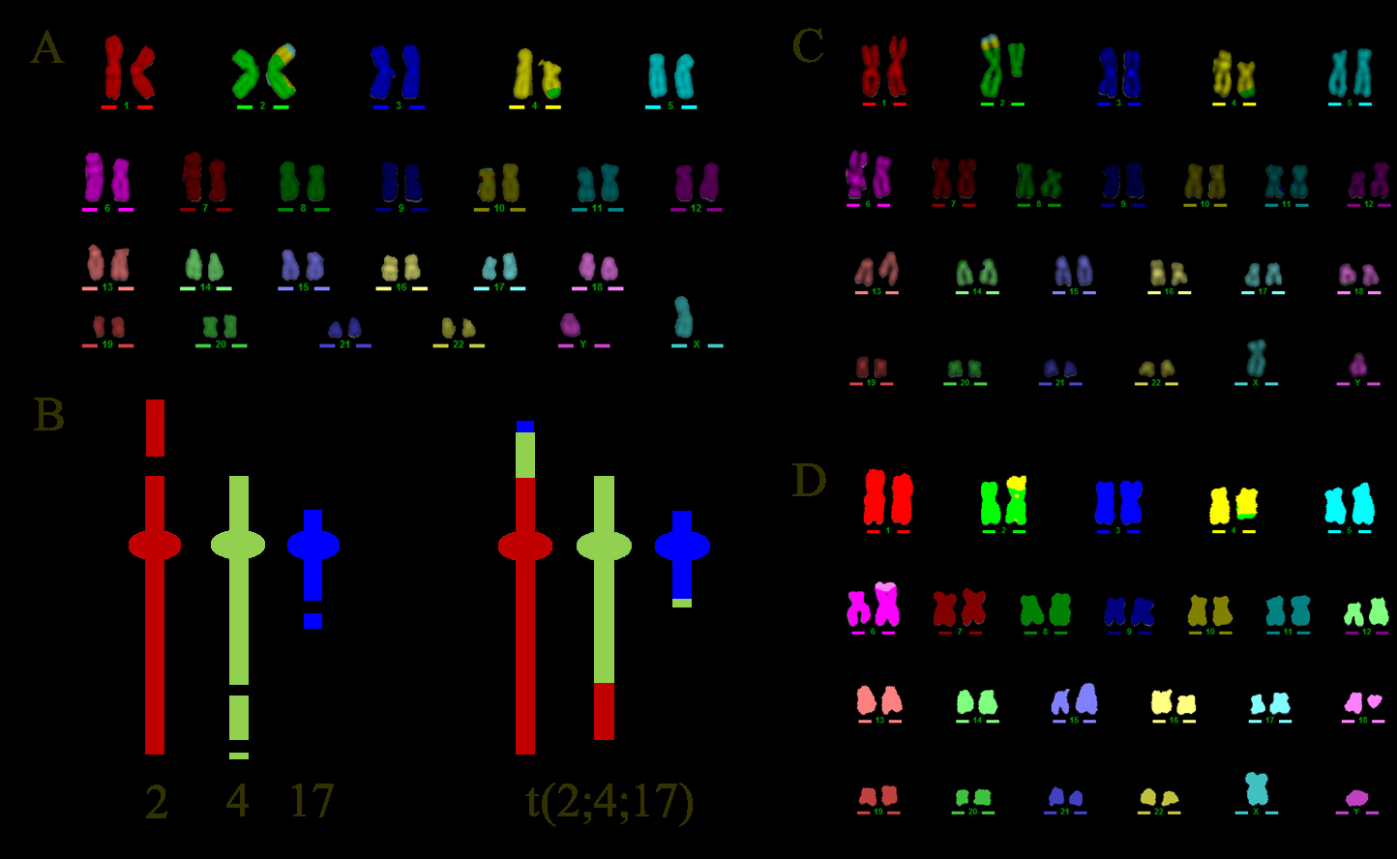
M-FISH karyotype of clonal complex exchange. M-FISH karyotype (A) and cartoon (B) showing the clonal complex exchange t(2;4;17) that was observed Td populations previously exposed to 0.5 Gy α-particles. The minimum number of chromosome breaks required to form the observed exchange are shown. Representative M-FISH karyotypes of the clonal complex that also contain additional non-clonal abnormalities such as chromosome breaks (del(2q) (B)) and simple translocations (t(6;18) (C)).

Both complex and simple exchange aberrations were detected in the descendants of α-particle irradiated cells (donor 2; Table 1). In all cases the exchanges were classified as being of the stable-type and theoretically capable of long-term transmission through cell division. Interestingly we observed a clonal complex rearrangement that consisted of a 3-way translocation between chromosomes 2, 4 and 17 (Table 1, Fig 3) in 58 cells on day 15, 16% of which contained additional non-clonal damage, for instance non-clonal translocations and chromatid breaks (Table 2). The frequency of this clonal complex aberration declined with cell culture such that it was observed (solely or together with additional damage) in only 4 cells on day 20, and 0 cells by day 27 (Tables 1 and 2).

Based on aberration frequencies observed in sham irradiated populations, chromosome break levels in α-particle exposed Td cells did not exceed background (0.045 and 0.059 for donors 1 and 2 respectively), bar a transient increase on day 20 in donor 2 (rising from 0.027 on day 15 to 0.091 and dropping again on day 27 to 0.011) (Table 3). By contrast, the chromatid break frequency in α-particle irradiated populations was elevated relative to sham (0.042 to 0.196 at day 35 for donor 1, and 0.078 to 0.122 by day 27 in donor 2 (Table 3).

In α-particle irradiated cells containing chromosome or chromatid breaks, multiple aberrations in the same metaphase were rarely observed in donor 1 (6%). However, additional damage was present in 43% of donor 2 cells. Specifically, chromosome breaks were observed singly (60%), in combination with another chromosome break (20%), and within cells containing a clonal complex aberration (13%). In addition, chromatid breaks were observed singly (59%), in combination with additional chromatid damage, or within cells already containing a clonal complex aberration (19%, of which 5% comprises >1 chromatid break).

### Aberrations in bone marrow and mature T-lymphocytes (Tw/Tp)

M-FISH analysis was carried out on Tw metaphase cells on days 15 and 20 post-irradiation. No exchange type aberrations were observed in sham-irradiated populations (Table 4), while background chromosome/chromatid break frequencies of 0.020/0.041 and 0.040/0.040 were observed in donor 1 and donor 2 respectively (Table 5).

**Table 4:** Chromosome exchange aberrations observed by M-FISH in undepleted and mature T-cell bone marrow mononuclear cells exposed to α-particle irradiation.

Exposure	Sample (days)	Total cells	% cells damaged	*f* Complex (no. of cells)		*f* Simple (no. of cells)	
				non-clonal	clonal	non-clonal	clonal
**Donor 1**							
**Tw**							
**Sham**	d15	49	8	0	0	0	0
**0.5 Gy α-particles**	d15	78	19	0.026 (2)	0	0.026 (2)	0
**Donor 2**							
**Tw**							
**Sham**	d15	50	10	0	0	0	0
	d20	25	8	0	0	0	0
**0.5 Gy α-**	d15	167	11^a^	0.018 (3)	0.006 (2)	0.006 (1)	0.006 (3)
**particles**	d20	103	6	0	0	0.010 (1)	0
**Tp**							
**0.5 Gy α-particles**	d15	225	9	0.004 (1)	0	0.009 (2)	0

^a^ includes clonal aberration scored as 1 cell to ascertain frequency

**Table 5:** Occurrence of non-clonal break aberrations in undepleted and mature T-cell bone marrow mononuclear cells exposed to α-particle radiation.

Exposure	Sample (days)	Total cells	Number of breaks (*f*)	
			chromosome	chromatid
**Donor 1**				
**Tw**				
**Sham**	15	49	1 (0.020)	2 (0.041)
**0.5 Gy α-particles**	15	78	9 (0.115)	2 (0.026)
**Donor 2**				
**Tw**				
**Sham**	15	50	0	5 (0.100)
	20	25	1^a^ (0.040)	1 (0.040)
**0.5 Gy α-**	15	167	5^a^ (0.030)	3 (0.018)
**particles**	20	103	3 (0.029)	2 (0.019)
**Tp**				
**0.5 Gy α- particles**	15	225	10^a^ (0.044)	6 (0.027)

^a^ includes clonal aberration scored as 1 cell to ascertain frequency

In α-particle irradiated Tw cells, both complex and simple exchanges were observed for both donors (Table 4). Nearly all (5 out of 6) complex aberrations observed were classified as being potentially transmissible. One non-transmissible complex exchange was observed in donor 1 Tw cells on day 15. The size and complexity of this aberration (involving 5 chromosomes and 9 breaks) was greater than any of the transmissible complex aberrations in this study and most likely represents a delayed 1^st^ division cell. The complex exchanges detected in donor 2 on day 15 (frequency 0.024, representing 5 cells, 2 containing the clonal complex t(1;5;10;19)), were reduced to undetectable levels by day 20. Similarly, the simple aberration frequency also declined over this time from 0.012 on day 15 (representing 4 cells, 3 belonging to one clone 46,XY,t(8;11),t(15;20)) to 0.010 (representing 1 cell) on day 20 post-irradiation. It is of interest to note that no newly arising aberrations were observed within either of these clones (Table 6).

**Table 6:** Evolution of sham and α-particle induced clonal aberrations with time in undepleted and mature T-cells.

Exposure	Sample (days)	Total cells (clone)	Clonal karyotype [no. of cells with karyotype]	Evolved karyotype
**Donor 1**	**Tw**			
**Sham**	d15	49 (0)		
**0.5 Gy α-particles**	d15	78 (0)		
**Donor 2**	**Tw**			
**Sham**	d15	50 (0)		
	d20	25 (1^a^)	46,XY, del(1)(q2-qter) [1]	
**0.5 Gy α-particles**	d15	167 (2)	46,XY,t(1;5;10;19)(p;p1q3;p14;q1) [2]	
		(3)	46,XY,t(8;11)(q24;p1),t(15;20)(q2;q13) [3]	
		(1^a^)	46,XY, del(1)(q2-qter) [1]	
	d20	103 (0)		
	**Tp**			
	d15	225 (0)		

^a^ clonal damage present in both sham and α-particle samples

Chromosome and chromatid break frequencies in α-particle irradiated Tw cells remained below background levels observed in sham irradiated populations, with the exception of chromosome breaks in donor 1 on day 15 (0.115 versus 0.020 in sham irradiated cells) (Table 5). Nearly half of the metaphases carrying chromosome breaks in donor 1 contained additional damage, in contrast to sham-irradiated and donor 2 α-particle irradiated cells (43% versus 0 and 0 respectively). Of these, 29% were observed with another chromosome break, and 14% with a chromatid break.

Tp populations were assessed at a single time-point (day 15) from donor 2 following α-particle exposure. Complex and simple exchange frequencies were 0.004 and 0.009 of total cells analysed respectively (Table 4). All exchange aberrations were classified as being potentially transmissible and no clones were observed (Table 6). Chromosome and chromatid break frequencies following α-particle exposure remained at background levels (0.044 and 0.027 respectively), no higher than those of sham-irradiated Td cells (0.059 and 0.051 respectively) (Table 5).

## Discussion

The applicability of complex chromosome aberrations as long-term indicators of previous high-LET α-particle exposure is dependent both on the induction of complex exchanges in haemopoetic stem/progenitor (HSC) cells and their subsequent transmission through lymphopoiesis. To assess this, we first optimised an extrathymic culture system that supported the differentiation of new T-cells from lymphoid stem and progenitor cells *in vitro* [35]. We then used this culture system to investigate the transmissibility of α-particle-induced complex aberrations through the lymphoid cell lineage.

T-cell receptor excision circles (TRECs) are often used as markers of naïve T-cell production, being produced just before lymphocytes become fully mature when the T-cell receptor locus is rearranged for expression [37]. Lacking a replication origin, TRECs are quickly lost on cell division, thereby providing evidence for recent lymphopoiesis in dividing cells [38, 39]. To validate that new mature T-lymphocyte populations in these experiments derive from α-particle irradiated progenitor cells rather than from residual T-cells post-depletion, TREC levels in Td populations were investigated throughout differentiation culture to day 12. Results suggest *de novo* T-cell production, with TREC numbers increasing from 0 on day 9 to 381.5 copies/10^5^ cells on day 12. Differentiation through the lymphoid lineage is also supported by the transient increase in CD4/CD8 expression (associated with an intermediate stage of lymphoid development), prior to the appearance of mature CD3^+^ cells in Td culture [40]. The majority of T-cells in Tw and Tp populations are CD8^+^ (70–88%) but in Td populations they are predominantly CD4^+^ (91% co-expression with CD3^+^). Therefore, it is unlikely that Td T-cell repletion has derived from the simple expansion of pre-existing BMNC T-lymphocytes. Comparisons between Td and Tw/Tp M-FISH data further corroborate *de novo* lymphopoiesis in Td culture. The overall frequency of complex clonal rearrangements detected in donor 2 was higher in Td compared to Tw/Tp samples, with the Td clone representing 0.265 and the Tw clone 0.012 of total cells analysed on day 15, and 0.040 and 0 respectively on day 20 (no Tp clone was observed at either time-point). This suggests expansion of a smaller α-particle irradiated population in Td compared to the larger populations of pre-existing mature T-cells in Tw/Tp samples.

We have shown previously that the traversal of a single α-particle through a spherical cell nucleus will predominantly result in the induction of a single complex exchange and more specifically, that the complexity of exchange observed by 24-colour painting techniques (M-FISH) is a reflection of the incident LET, qualitative structure of the radiation track and the geometric organization of the cell nucleus irradiated [28, 29, 41–43]. The data generated in this present study are consistent with this. Although the full spectrum of α-particle-induced damage was not assessed in hierarchical lymphocyte progenitors on day 0, transmissible complex exchanges, entirely equivalent to those previously observed in PBL and CD34^+^ cells (based on number of breaks and chromosomes involved) were classified by M-FISH 15 days after exposure of Td, Tp and Tw cell populations to 0.5 Gy high-LET α-particles. Typically, these complex rearrangements involved the mis-repair of a minimum of 3 to 5 breaks in 3 or 4 different chromosomes. Thus, the persistence of complex exchanges through several generations of lymphoid lineage differentiating cells into mature T-cells supports their usefulness as long-term indicators of past α-particle exposure.

The frequency of clonal exchange aberrations was observed to decrease with time in culture, presumably due to dilution of these clones with ongoing proliferation and possibly also to the occurrence of newly arising aberrations instigating apoptosis associated with genetic instability [44]. *In vivo*, the circulatory lymphocyte pool is continually replenished from the HSC compartment [45], meaning a similar dilution of observable exchanges will occur overtime however a proportion of new PBL will presumably be derived from surviving lymphoid progenitors, a proportion of which will contain stable complex exchanges [24, 46–48]. In addition to this, since T-lymphocyte populations are normally quiescent, directly exposed PBL, for instance in the lymph nodes, are unlikely to be lost due to mitotic catastrophe meaning it is probable that complex exchanges of both the stable and unstable type may be detected in the peripheral blood after *in vivo* exposure. Indeed PBL sampled from retired workers chronically exposed to internalised plutonium (α-particle emitter) contained complex chromosome exchanges at a frequency of 0.02–0.08, of which ~20–50% were classified as transmissible most likely resulting from dose to the red bone marrow [22]. The remaining non-transmissible complexes are proposed to represent mature T-cells which remain in their 1^st^ interphase for months or even years after irradiation, until sampled and artificially stimulated to divide in culture. The biological mechanism for this long-term persistence of such heavily damaged PBLs is unclear however similar observations have been reported elsewhere [24, 49, 50]. Thus, complex chromosome exchanges, irrespective of their stability through cell division, are reliable and useful indicators of chronic α-particle exposure.

The induction of simple exchanges i.e. dicentrics and translocations by low-LET radiation is well characterized, indeed their presence in blood lymphocytes can be used to estimate radiation dose [51, 52]. Simple translocations, which are capable of long-term transmission, are also induced after α-particle exposure in a manner that is strongly influenced by both the incident LET and the organization of the genome intersected by the α-particle track [25, 41]. Specifically, the proportion of simple:complex exchanges in spherical cells will increase with decreasing LET of the traversing particle [29]. Therefore, simple translocations will be directly induced in lymphoid progenitor cells and will be capable of populating the circulatory PBL pool. In addition to this, the background translocation frequency is known to increase with age according to individual genetic susceptibility and lifestyle [53, 54], including stem and progenitor cells of the haemopoietic system. Accordingly translocations formed by both of these mechanisms would be clonal in nature [55]. In this study, clones containing simple exchanges were observed that can be accounted for by the above mentioned mechanisms, specifically we observed a clone containing two translocations in Tw cells after exposure to a fluence of 1 α-particle/cell while a pre-existing simple exchange was observed in both sham and irradiated donor 1 cells. In contrast to this, we also observed new, non-clonal reciprocal translocations to arise in α-particle exposed Td cells indicative of ongoing genomic instability.

The *de novo* generation of chromosome and chromatid aberrations in descendants of α-particle irradiated HSC has been demonstrated in both humans and mice [56–60]. In the Td populations analysed here, an increase in the frequency of chromatid aberrations is evident in the progeny of HSC and lymphoid progenitors by day 35 (donor 1) and day 27 (donor 2) post-irradiation (rising from 0.010 on day 27 to 0.196 on day 35, and from 0.040 on day 20 to 0.122 by day 27 respectively) and a ~1:1 ratio of non-clonal chromatid:chromosome aberrations was observed within Td cells carrying clonal chromosomal damage. Typically, break aberrations result in a loss of genetic material and are therefore not transmissible through cell division. Thus, the presence at all time-points analysed of chromosome breaks both with and without associated acentric fragments implies that these aberrations are being continuously generated and lost throughout the culture period [61]. This is substantiated by the presence of non-clonal chromosome breaks within clonal Td cells. Simple exchanges can also form part of an instability phenotype and interestingly we observed two *de novo* simple translocations in Td cells carrying a clonal complex exchange. In other words, an expression of genomic instability was observed in the progeny of cells that show evidence of previous α-particle exposure and also in cells that show no such marker. These observations may support the opinion that radiation-induced genomic instability may not necessarily reflect genomically unstable cells *per se* but rather cellular responses to ongoing damage, for instance from ROS or inflammatory signals [62–64]. No corresponding instability effect was observed in the progeny of α-particle exposed mature T-cell populations (Tw/Tp), although later time-points (past day 20) for this population would have enabled direct comparison with Td results. This lack of instability corresponds with findings from previous analysis of mature T-cells ~20 population doublings following α-particle irradiation [33] but is in contrast to Kadhim *et al*. 2001 who were able to demonstrate an instability phenotype in 25% of irradiated PBL progeny after just 13 population doublings [65].

Genetic factors are known to play an important role in determining individual responses to DNA damage and can considerably impact instability levels observed (Kadhim and Wright 1998, Watson *et al*. 1997, Limoli *et al*. 2000). In this study although both donors showed evidence of α-particle exposure (non-clonal (donor 1) and clonal/non-clonal (donor 2) complexes in Tw populations) and evidence for α-particle-induced delayed instability (increased frequencies of chromatid aberrations in Td populations), only donor 2 showed evidence for the non-clonal evolution of a clonal karyotype and only then, within the Td population. This is clearly of interest and may reflect how variations in genetic background influence karyotypic stability. In this scenario however the lack of complex aberrations in donor 1 Td cells is most likely a reflection of the low transmissibility of α-particle-induced complexes and the limited number of cells examined. Investigation of a wider donor pool should help clarify this.

Studies based on the regeneration of ablated haemopoietic systems from α-particle irradiated HSC have confirmed the formation of unstable karyotypes in clonal cells over prolonged periods *in vivo* [59, 60, 66]. Our observations extend this by demonstrating evidence of genomic instability in cells containing a historical cytogenetic marker of direct intersection of an α-particle track *in vitro* and has implications not just for understanding the relationship between α-particle exposure and carcinogenesis but also for the emerging use of ^223^Ra for the treatment of bone metastatic prostate and breast cancers [6, 7]. For instance, the identification of characteristic complex aberrations in peripheral blood may inform on dose to the marrow providing data for treatment planning and secondly, it may provide important information on risks of second, therapy-related, cancers.

In conclusion, this study used the extra-thymic T-cell differentiation of human haemopoietic stem and progenitor cells to assess the transmission of chromosomal damage initially-induced after exposure to high-LET α-particles (at a fluence of 1 α-particle per cell). Our findings support the usefulness and reliability of employing complex chromosome exchanges as indicators of past or ongoing exposure to high-LET radiation and broaden the potential applicability to include the identification of individuals exposed to radiation of unknown or mixed radiation exposures, in addition to evaluating medical applications of α-emitting radionuclides in advancing radiotherapeutic strategies.
